# Unveiling the Structural Behavior under Pressure of Filled M_0.5_Co_4_Sb_12_ (M = K, Sr, La, Ce, and Yb) Thermoelectric Skutterudites

**DOI:** 10.1021/acs.inorgchem.1c00682

**Published:** 2021-04-26

**Authors:** João Elias F. S. Rodrigues, Javier Gainza, Federico Serrano-Sánchez, Mateus M. Ferrer, Guilherme S. L. Fabris, Julio R. Sambrano, Norbert M. Nemes, José L. Martínez, Catalin Popescu, José A. Alonso

**Affiliations:** † Instituto de Ciencia de Materiales de Madrid, CSIC, Cantoblanco, E-28049 Madrid, Spain; ‡ European Synchrotron Radiation Facility, ESRF, 71 Avenue des Martyrs, 38000 Grenoble, France; § CCAF, PPGCEM/CDTec, Federal University of Pelotas, CEP 96010-610 Pelotas, Rio Grande do Sul, Brazil; ∥ Materials Science and Engineering Postgraduate Program, Department of Materials Engineering, Federal University of Rio Grande do Norte, 59078-970 Natal, Brazil; ⊥ Modeling and Molecular Simulation Group, São Paulo State University, 17030-360 Bauru, SP, Brazil; # Departamento de Física de Materiales, Universidad Complutense de Madrid, E-28040 Madrid, Spain; ∇ CELLS−ALBA Synchrotron, E-08290 Cerdanyola del Valles, Barcelona, Spain

## Abstract

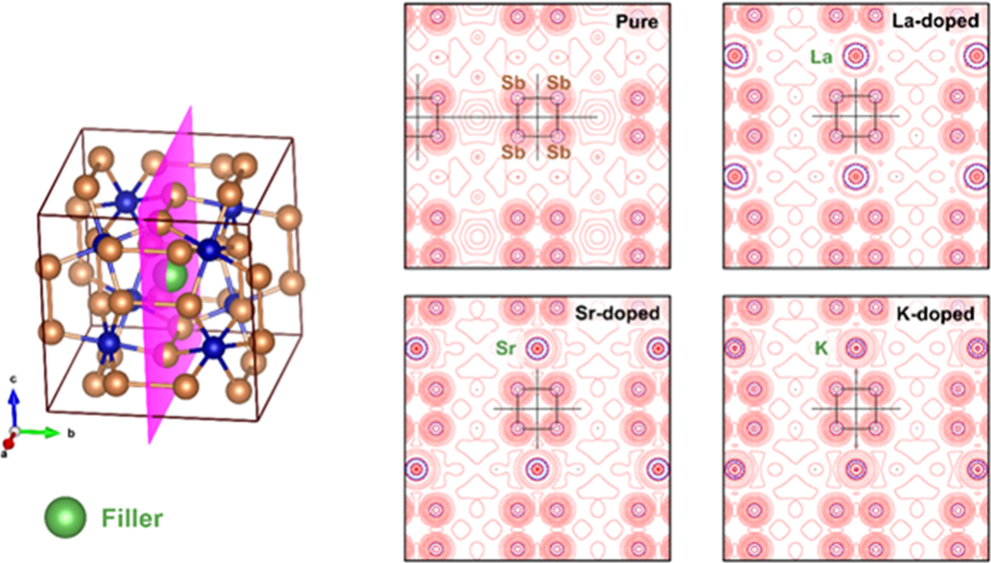

Skutterudite-type compounds based on □Co_4_Sb_12_ pnictide are promising for thermoelectric application due to their good Seebeck values and high carrier mobility. Filling the 8*a* voids (in the cubic space group *Im*3̅) with different elements (alkali, alkali earth, and rare earth) helps to reduce the thermal conductivity and thus increases the thermoelectric performance. A systematic characterization by synchrotron X-ray powder diffraction of different M-filled Co_4_Sb_12_ (M = K, Sr, La, Ce, and Yb) skutterudites was carried out under high pressure in the range ∼0–12 GPa. The isothermal equations of state (EOS) were obtained in this pressure range and the Bulk moduli (*B*_0_) were calculated for all the filled skutterudites, yielding unexpected results. A lattice expansion due to the filler elements fails in the description of the Bulk moduli. Topochemical studies of the filler site environment exhibited a slight disturbance and an increased ionic character when the filler is incorporated. The mechanical properties by means of Bulk moduli resulted in being sensitive to the presence of filler atoms inside the skutterudite voids, being affected by the covalent/ionic exchange of the Co–Sb and Sb–Sb bonds.

## Abstract 2

High-pressure studies in the range ∼0−12 GPa were carried out for M-filled Co_4_Sb_12_ (M = K, Sr, La, Ce, and Yb) skutterudites. The isothermal equations of state (EOS) were obtained and the Bulk moduli (*B*_0_) were calculated, yielding unexpected results. Topochemical studies of the filler site environment exhibited a slight disturbance and an increased ionic character when the filler is incorporated. (left) 3D structure and (right) Laplacian of electronic density isolines of the M−Sb and Sb−Sb interactions.

## 1 Introduction

In the field of energy conversion technologies, thermoelectric materials have been showing interesting applications in recovering electrical energy from temperature gradients. Well-designed devices can integrate a waste heat recovery system or solid-state-based cooling/heating with no moving parts and long-term stability. Indeed, due to the Peltier effect, these materials can be used in a thermoelectric cooler in several practical applications such as small refrigerators for beverages, CPU cooling, vaccine refrigeration for portable bags, thermal stabilization devices for lasers or CCD detectors, etc. For a better performance, a high efficiency should be achieved through a figure of merit (*zT* = *S*^2^σ*T*/κ_T_) above 1.^1^ This dimensionless parameter has a threefold dependency: the Seebeck coefficient (*S*), carrier electrical conductivity (σ), and thermal conductivity (κ_T_). A good thermoelectric material should balance these three physical quantities in order to provide a reasonable performance for applications.

Among the thermoelectric compounds, the binary skutterudites □T_4_X_12_ (T = Co, Rh, or Ir; X is a pnictogen element) depict interesting Seebeck values and high carrier mobility; however, the thermal conductivity (typically around 10 Wm^–1^ K^–1^) is not so attractive to enhance a value of *zT* > 1, mainly due to the strong covalent bonding of the framework (TX_3_)_4_^3–^. Such a structure has open cavities (voids: □), which can be filled by guest atoms, thus producing a material with phonon-glass electron-crystal (PGEC) properties.^2,3^ As a result, the thermal conductivity might be reduced by an order of magnitude, as the phonon mean free path is decreased due to the presence of fillers. The local bonding of the filler inside the cavities plays a pivotal role for enhancing the *zT* parameter. Rare-earth elements have been usually a common choice to fill these voids, with plenty of examples reported in the literature.^4−10^ Recently, our group demonstrated that the high-pressure synthesis is an effective tool to stabilize lanthanides into the voids of the □Co_4_Sb_12_ skutterudite,^11^ as in the case of La,^12^ Ce and Yb,^13^ and mischmetal,^14^ consequently reducing the thermal conductivity (<2–3 Wm^–1^ K^–1^).

Under the application of an external high-pressure, some interesting features were observed in filled skutterudites, including a metal–insulator transition in PrFe_4_P_12_ at 2.4 GPa and an insulator–metal transition in PrRu_4_P_12_ at 12 GPa.^15^ Another example is the filling fraction in CoSb_3_ skutterudites, which is enlarged with increasing the applied pressure,^16^ and sometimes, the high-pressure synthesis is the only procedure that can be used to introduce a certain filler element.^17,18^ □Co_4_Sb_12_ presented self-insertion reactions, in which the transition □Co_4_Sb_12_ → Sb*_x_*Co_4_Sb_12-*x*_ takes place under high pressure with no volume discontinuity.^19^ The elastic properties of filled skutterudites may unveil details on the local bonding of the fillers inside the voids and, therefore, useful insights into the features of PGEC systems.

Herein, we provide a structural investigation by synchrotron radiation in unfilled □Co_4_Sb_12_ and filled M_0.5_Co_4_Sb_12_ (M = K, Sr, La, Ce, and Yb) skutterudites, previously synthesized under high-pressure conditions (3.5 GPa) and studied also under compression with pressures up to 12 GPa. The isothermal equations of state (EOS) were determined in this pressure range, and the Bulk moduli (*B*_0_) were obtained for all the filled skutterudites. Computational calculations by means of density functional theory were also conducted in order to give light about the filler local environment and involved chemical interactions. We aim to correlate the lattice expansion due to the filler atoms with the Bulk moduli experimentally derived and possible consequences from a local point of view.

## 2 Experimental Section

### 2.1 High-Pressure Synthesis

Pure Co_4_Sb_12_ and filled skutterudites of nominal composition M_0.5_Co_4_Sb_12_ (M = K, Sr, La, Ce, and Yb) were synthesized under high pressure (HP) at moderate temperatures in a piston-cylinder press (Rockland Research Co). Stoichiometric amounts of M, Co, and Sb were mixed and ground in a N_2_-filled glove box to avoid surface oxidation. Ternary elements M were taken from metallic powders, except K which was extracted from KH (Alfa Aesar, 35% in mineral oil). The mixture of 1.2 g was sealed in a niobium capsule of 5 mm in diameter and, then, introduced inside a cylindrical graphite heater. The chemical reaction occurred under 3.5 GPa for 1 h at 800 °C; then, the sample was quenched and the pressure was released. The skutterudite phase formation was confirmed by laboratory X-ray diffraction (Cu-Kα radiation; λ = 1.54053 Å). Details on the synthesis and room condition structural properties of HP-synthesized M_0.5_Co_4_Sb_12_ can be found elsewhere.^11−14,20^

### 2.2 High-Pressure Characterization

High-pressure investigations were performed in powder samples on the high-pressure/microdiffraction station of the BL04-MSPD beamline^21^ of the CELLS-ALBA synchrotron (Barcelona, Spain), selecting an incident beam at Cd *K*-edge (λ = 0.4642 Å). Two diamond anvil cells with diamond culet sizes ranging from 500 to 700 μm were used; the pressure chambers were 200 and 300 μm hole drilled in a 50 μm-thick pre-indented Inconel gasket. Silicone oil was used as pressure transmitting medium: “Rhodorsil 47V1000” commercialized by VCR.^22^ Grains of copper were placed inside the pressure cavity and used as the pressure sensor through copper EOS.^23^ The two-dimensional X-ray patterns were recorded using a Rayonix CCD detector. The sample–detector distance (180 mm) and the beam center position were calibrated from LaB_6_ diffraction data measured under exactly the same conditions as the sample. The Debye–Scherrer rings formed were integrated using the DIOPTAS software.^24^ The as-obtained patterns were refined by the Rietveld method in the framework of FULLPROF suite.^25^ The following parameters were refined: zero-point error, background coefficients, scale factor, asymmetry correction factors, lattice parameters, atomic coordinates, and isotropic displacements.

### 2.3 Computational Details

The theoretical models were performed according to density functional theory (DFT) with PBE functional,^26^ implemented in the CRYSTAL17 package.^27^ The cobalt, antimonium, and potassium atomic centers were defined by the POB-TZVP basis set developed by Bredow and co-workers.^28−30^ Lanthanides, potassium, and strontium atomic centers were defined by the effective core potential basis set developed by Maier and co-workers.^31^

The Coulomb and exchange series were controlled by a set of five thresholds (10^–8^, 10^–8^, 10^–8^, 10^–8^, and 10^–16^), which represent the overlap and penetration for Coulomb integrals, the overlap for HF exchange integrals, and the pseudo-overlap, respectively. The used shirking factors were 6 and 6 for Pack–Monkhorst and Gilat net, respectively. The structure optimization convergence was achieved on gradient components and nuclear displacements with tolerances on their root mean square set to 0.0003 and 0.0012 a.u., respectively.

The bonds of the different models were analyzed by “Quantum Theory: Atoms in Molecules” (QTAIM), which provides a quantum description of the electron’s characteristics in the chemical bonds. The QTAIM analysis was carried out with the TOPOND program within the CRYSTAL17 package.^32^ The crystalline models were represented by VESTA software.

## 3 Results and Discussion

**Figure 1:**
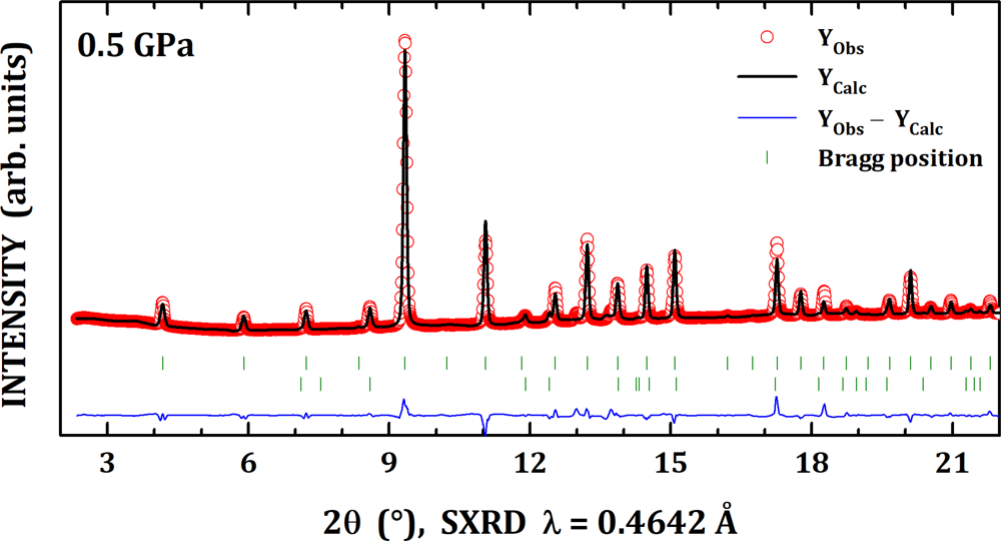
Near room condition synchrotron X-ray diffraction pattern of □Co_4_Sb_12_ skutterudite. The second series of Bragg positions corresponds to Sb metal minor impurity.

**Figure 2:**
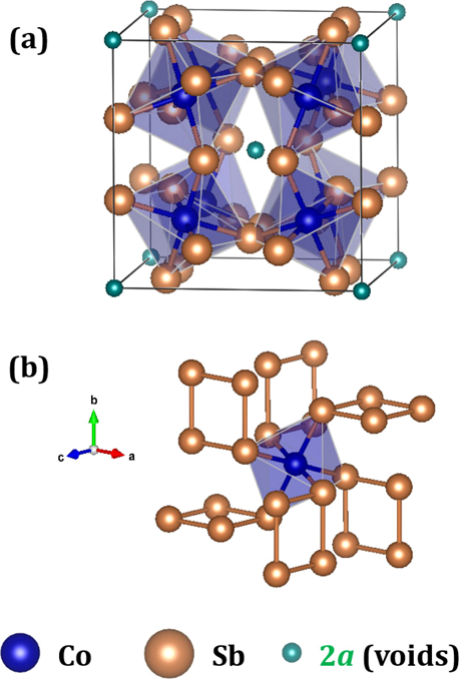
Sketch of the crystal structure of the filled skutterudite with guest atoms at 2*a* Wyckoff positions (a). View of the configuration of [Sb_4_] rings that exist in such a structure (b).

High-pressure synchrotron X-ray diffraction measurements were carried out up to ∼12 GPa for the skutterudite compounds with the following nominal compositions: □Co_4_Sb_12_, K_0.5_Co_4_Sb_12_, Sr_0.5_Co_4_Sb_12_, La_0.5_Co_4_Sb_12_, Ce_0.5_Co_4_Sb_12_, and Yb_0.5_Co_4_Sb_12_. Figure 1 shows the near room condition X-ray diffraction pattern of □Co_4_Sb_12_, as refined according to the body-centered space group *Im*3̅ (*N*° 204, *T_h_*^5^) with eight formula per unit cell (*Z* = 8). The atomic distribution in this structure is the following: Co atoms are at 8*c* Wyckoff sites (^1^/_4_, ^1^/_4_, ^1^/_4_) and the Sb ones are positioned at 24*g* sites (0, *y*, *z*). Indeed, the *y* and *z* fractional coordinates and the lattice constant *a* are the only three parameters for representing the skutterudite crystal structure.^33^ As depicted in Figure 2a, the skutterudite consists of a network of strongly tilted [CoSb_6_] octahedra sharing corners, forming large cages centered at 2*a* positions (0, 0, 0). These voids, represented by □, may be filled by guest atoms. For pristine □Co_4_Sb_12_, a minor impurity of Sb metal in a fraction of 6.1% was found after the refinement of the scale factors of both phases.

**Figure 3:**
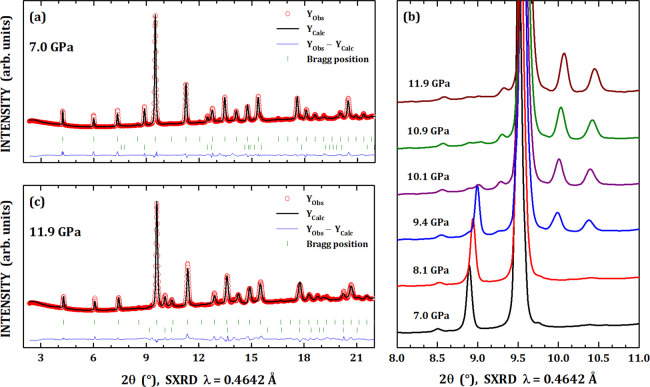
Synchrotron X-ray diffraction patterns of □Co_4_Sb_12_ under 7.0 GPa (a) and 11.9 GPa (c). Details on the pressure evolution of Sb metal secondary phase (b), showing its phase transition from a rhombohedral (S.G. *R*3̅*m*) to close-packed hexagonal (S.G. *P*6_3_/*mmc*) phase around 9.4 GPa.

Under high-pressure conditions up to 12 GPa, the □Co_4_Sb_12_ system did not exhibit any signal of structural phase transition, for instance, see the Rietveld refined pattern under 7.0 GPa in Figure 3a. Some details on the pressure evolution of the secondary minor impurity of Sb metal are shown in Figure 3b. In particular, the Sb metal has a rhombohedral unit cell (S.G. *R*3̅*m*) under room conditions, which undergoes two successive transitions to cubic (7.0 GPa) and hexagonal (8.5 GPa) phases.^34^ However, in this work, only the hexagonal phase was identified under high pressure and starting at 9.4 GPa. In Figure 3c, the room-condition Sb rhombohedral phase was replaced by the close-packed hexagonal structure (S.G. *P*6_3_/*mmc*) in order to refine the synchrotron X-ray pattern under 11.9 GPa. It is clear that the transition takes place at 9.4 GPa and the hexagonal phase is completely stabilized at 10.1 GPa.

Although previous works in the literature already reported the isothermal equation of state for pure □Co_4_Sb_12_, we also performed here a high-pressure study of this compound. In our case, the skutterudites were fabricated using HP synthesis at moderate temperatures. Therefore, it would be interesting to probe their structural behavior under high pressure. The main reason for that is the improved thermoelectric properties observed in HP-synthesized skutterudites in recent years.^11−14^ For example, the total thermal conductivity (κ_T_) at room temperature in □Co_4_Sb_12_ synthesized by conventional methods reaches values of 10 Wm^–1^ K^–1^, while those HP-prepared depict a κ_T_ of 4.3 Wm^–1^ K^-1,^^20^ based on the structural defects. It means that the HP condition has a pivotal role in enhancing the thermoelectric behavior in skutterudites.

**Figure 4:**
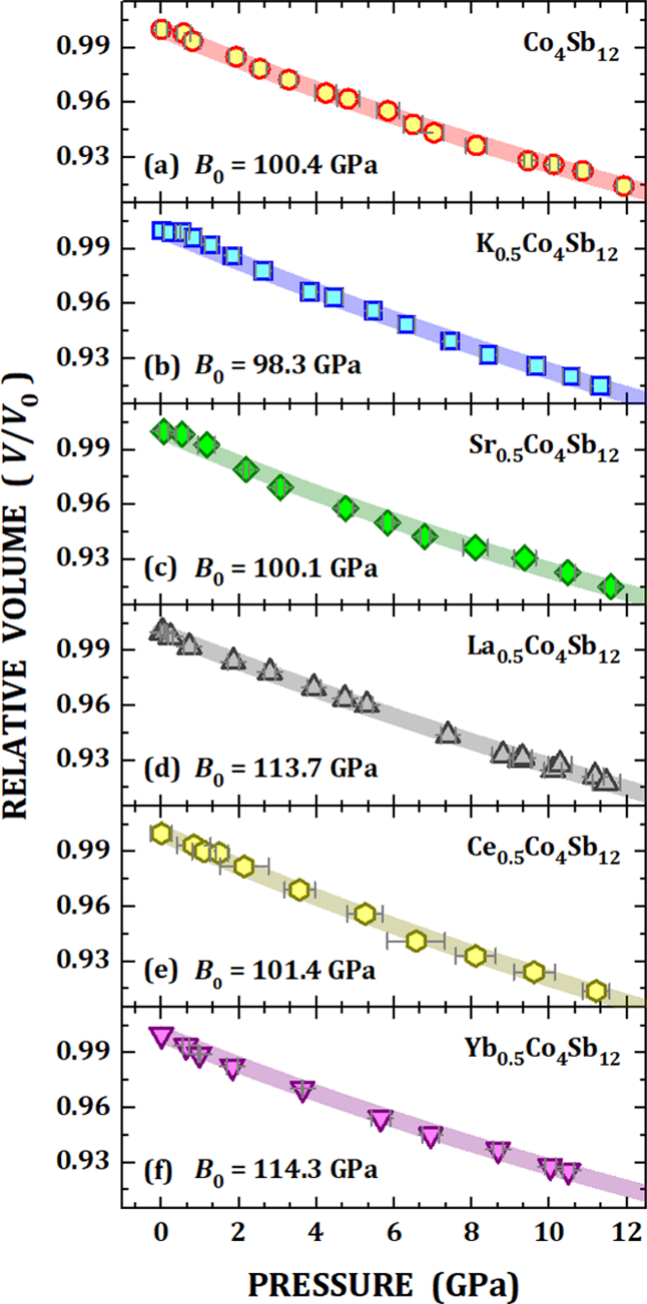
Relative volume (*V*/*V*_0_) as a function of applied pressure (open symbols) together with the third-order Birch–Murnaghan (BM) equation of state (EOS) fitting (thick lines). The fitting was performed for the unfilled □Co_4_Sb_12_ (a) and filled *M*_0.5_Co_4_Sb_12_ skutterudites, in which M = K (b), Sr (c), La (d), Ce (e), and Yb (f).

Figure 4a represents the evolution of the relative volume (*V*/*V*_0_) as a function of pressure for □Co_4_Sb_12_ within the interval from 0.5 up to 11.9 GPa. *V*_0_ stands for the unit-cell volume under atmospheric conditions, which was already known for each compound before the HP experiment (see in Table 1). One may notice that no discontinuity was detected in the unit-cell volume in such a pressure regime, which is the quasi-hydrostatic regime for silicone oil used here.^22^ The experimental data were adjusted to the third-order Birch–Murnaghan (BM) isothermal equation of state (EOS)^35^ as follows:
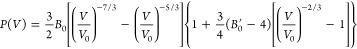
1which allows us to determine the Bulk modulus (*B*_0_) and its pressure derivative (*B*_0_′ = *∂B*_0_/*∂P*). In particular, the *B*_0_′ parameter was kept equal to 4. Table 1 summarizes the *B*_0_ value for □Co_4_Sb_12_ obtained from this work. The interesting result is that our □Co_4_Sb_12_ has a *B*_0_ of 100.4(3) GPa, which means that the HP synthesis makes the skutterudite more resistant to the compression when compared to the cobalt antimony prepared by conventional methods (*B*_0_ = 93.2(6)^36^). Also, it is even more resistant than the □Co_4_Sb_12_ prepared using a wedge-type cubic-anvil high-pressure apparatus (*B*_0_ = 81(1) GPa^37^).

**Table 1:** Lattice Constants under Room Conditions (*a*) and Bulk Modulus (*B*_0_) for Unfilled □Co_4_Sb_12_ and Filled M_0.5_Co_4_Sb_12_ (M = K, Sr, La, Ce, and Yb) Skutterudites

		this work						SXRD in refs^11−14^	
		2D detector			DFT			high angular resolution mode	
composition		*a* (Å)	*B*_0_ (GPa)		*a* (Å)	*B*_0_ (GPa)		*a* (Å)	
									
□Co_4_Sb_12_								□Co_4_Sb_11.6_	
		9.0358(8)	100.4		9.083	93.8		9.03588(2)	
									
K_0.5_Co_4_Sb_12_								K_0.13_Co_4_Sb_12_	K_0.11_Co_4_Sb_12_
		9.0559(3)	98.3		9.149	88.8		9.0459(1)	9.03917(8)
									
Sr_0.5_Co_4_Sb_12_								Sr_0.48_Co_4_Sb_12_	Co_4_Sb_12_
		9.0887(5)	100.1		9.139	92.9		9.0887(7)	9.0393(7)
									
La_0.5_Co_4_Sb_12_								La_0.17_Co_4_Sb_11.64_	La_0.052_Co_4_Sb_11.68_
		9.0508(5)	113.7		9.123	94.6		9.0545(8)	9.0405(8)
									
Ce_0.5_Co_4_Sb_12_								Ce_0.10_Co_4_Sb_11.64_	Ce_0.05_Co_4_Sb_11.64_
		9.0480(5)	101.4		9.130	91.4		9.04805(5)	9.03940(6)
									
Yb_0.5_Co_4_Sb_12_								Yb_0.26_Co_4_Sb_11.54_	Yb_0.06_Co_4_Sb_11.54_
		9.0512(5)	114.3					9.06186(4)	9.0444(1)
									

As pointed out earlier, the skutterudite structure can be viewed as eight subcubes cornered by Co atoms, with planar [Sb_4_] rectangles occupying six of these cubes (Figure 2b) and, then, leaving two void spaces for a guest atom (at 2*a* sites) (see in Figure 2a). Such a filler atom may affect the local bonding of [Sb_4_] units of Figure 2b and, therefore, the electronic band structure near the Fermi level.^33^ The amount *f* of guest atoms into the voids is usually limited by the filling fraction limit, which depends on the chemical nature of the fillers (such as its electronegativity^38^) and their cationic sizes. In the single-filled skutterudite M*_f_*Co_4_Sb_12_, the value of *f* barely reaches 0.25 for rare-earth elements (except for Eu with *f* = 0.45); for alkali and alkaline-earth metals, the filling fraction limit reaches *f* = 0.45 for K and 0.40 for Sr.^39^ In our previous works, we reported on the high-pressure synthesis and structural characterization of filled M_0.5_Co_4_Sb_12_ skutterudites;^11−14^ two conclusions were obtained: the HP condition followed by quenching leads to two coexisting filled phases with uneven filling fractions, and this coexistence contributes to the reduced thermal conductivity. Probably, the inhomogeneous distribution of pressure within the Nb capsule, in intergranular regions with respect to the bulk of the grains, leads to the mentioned filling fraction distribution. The reduction of the thermal conductivity is achieved by creating a new hierarchy at the nanoscale. In this way, the HP-synthesized skutterudites present an all-length-scale hierarchy atomic scale with fillers, nanoscale with coexisting phases, and mesoscale with grain boundary effects. This hierarchy enhances the phonon scattering, thus decreasing the thermal conductivity at a macroscopic level.

The coexisting phases were detected due to the splitting of synchrotron X-ray diffraction peaks at high scattering angles, being collected in high-angular resolution mode.^21^ For the diffraction experiments in high-pressure mode, the information on the coexisting phases was lost since there is one order of magnitude difference of angular resolution between the two modes. As a result, the peak splitting was not indeed detected, and the refinement only gives one single skutterudite phase for all the filled samples. On the other hand, the effect of filling could be detected by the lattice expansion (see in Table 1), which shows that the filler element entered into the skutterudite structure. Despite some differences, we concluded that the lattice constants collected under high pressure would, at least, represent the filled skutterudite phase with the greatest filling fraction reached for each filled M_0.5_Co_4_Sb_12_.

From a crystallo-chemical point of view, the guest atom tends to expand the unit-cell volume, depending on the filling fraction, and it would decrease the compressibility of filled skutterudites. In the literature, the opposite behavior was observed by Kraemer *et al.*(36) for LaFe_3_CoSb_12_ (*B*_0_ = 87.4(6) GPa) in comparison with the unfilled □Co_4_Sb_12_ (*B*_0_ = 93.2(6) GPa). However, such a compound has the effect of both Fe and Co atoms in the doped filled skutterudite structure. In our case, we aim to evaluate only the effect of chemical pressure generated by the guest atoms at 2*a* positions in binary □Co_4_Sb_12_. In this way, synchrotron X-ray diffraction studies under high pressure (0 up to 12 GPa) were performed in the following filled M_0.5_Co_4_Sb_12_ skutterudites, for M = K, Sr, La, Ce, and Yb. Different valence states 1+ (K), 2+ (Sr), and 3+ (La, Ce, and Yb) were chosen in order to provide more information on the charge transfer and its role in compressibility of filled skutterudites.

**Figure 5:**
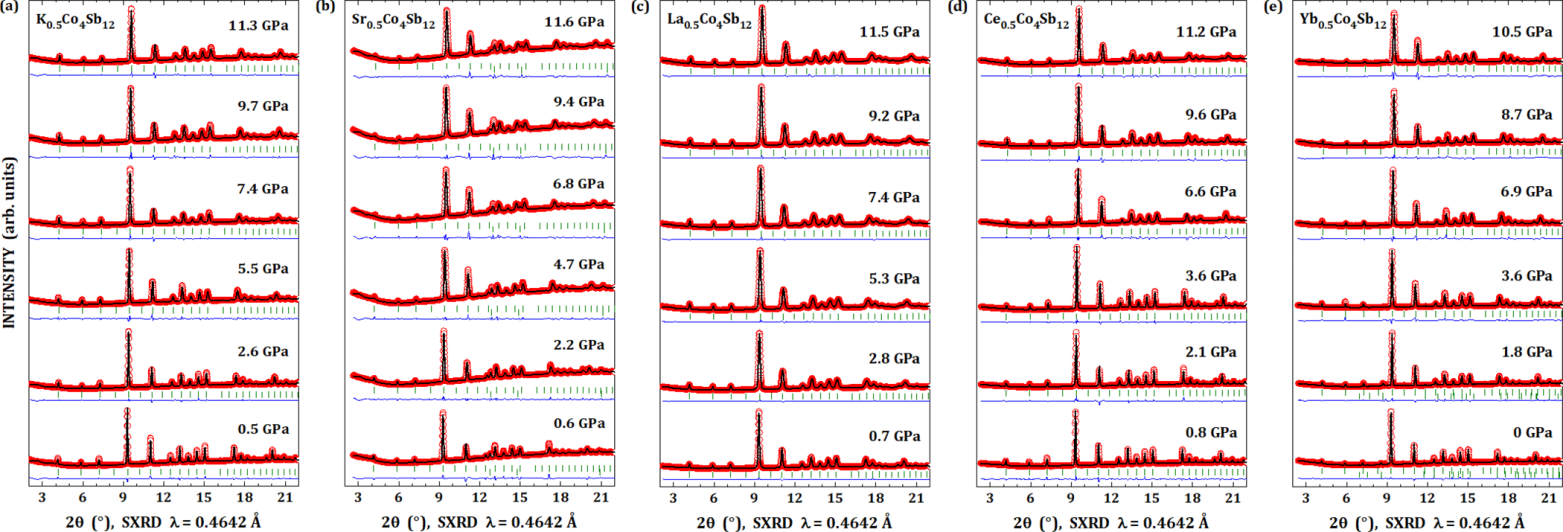
Synchrotron X-ray diffraction patterns of the filled M_0.5_Co_4_Sb_12_ skutterudites under selected pressures, in which M = K (a), Sr (b), La (c), Ce (d), and Yb (e). Red open circles represent the experimental data, the black line refers to the calculated profile, the blue line is the difference between experimental and calculated data, and dark green bars denote the Bragg reflections.

Figure 5 shows some refined patterns under selected pressures for each filler element, i.e., K (a), Sr (b), La (c), Ce (d), and Yb (e). No new peaks are observed in the diffraction patterns, although the reflection lines shift to high angle with increasing pressure, as expected. A good quality refinement was obtained for all the compounds in all the range of investigated pressures. The samples with K, La, and Ce atoms were refined using only a single skutterudite phase with the filler element at the 2*a* position, while for Sr and Yb, a second phase of Cu and Sb metals was introduced in the refinement, respectively. For all the samples, the Cu metal is a pressure sensor. Panels (b) to (f) of Figure 4 depict the evolution of the relative volume (*V*/*V*_0_) as a function of pressure up to 12 GPa for the filled skutterudites investigated in this work. The fitting by the third-order Birch–Murnaghan (BM) isothermal equation of state (EOS) revealed some changes in the Bulk modulus, as compared to the unfilled □Co_4_Sb_12_ (see in Table 1).

Theoretical models according to the DFT method were used to better understand the high-pressure behavior in the filled skutterudites. A filling fraction limit of *f* = 0.5 was considered for all the simulated filled samples: K, Sr, La, and Ce. The effect of hydrostatic pressure was simulated in unfilled □Co_4_Sb_12_ and filled compounds ranging from ∼0 up to 12 GPa. The lattice constants optimized for the calculations are shown in Table 1. Although perfect agreement was not reached, one may see the filler effect by expanding the lattice constant. The calculated relative volume (*V*/*V*_0_) as a function of pressure for each referred sample is displayed in Figure 4. Table 1 also includes the Bulk modulus from DFT calculations for the referred filled skutterudites investigated in this work.

**Figure 6:**
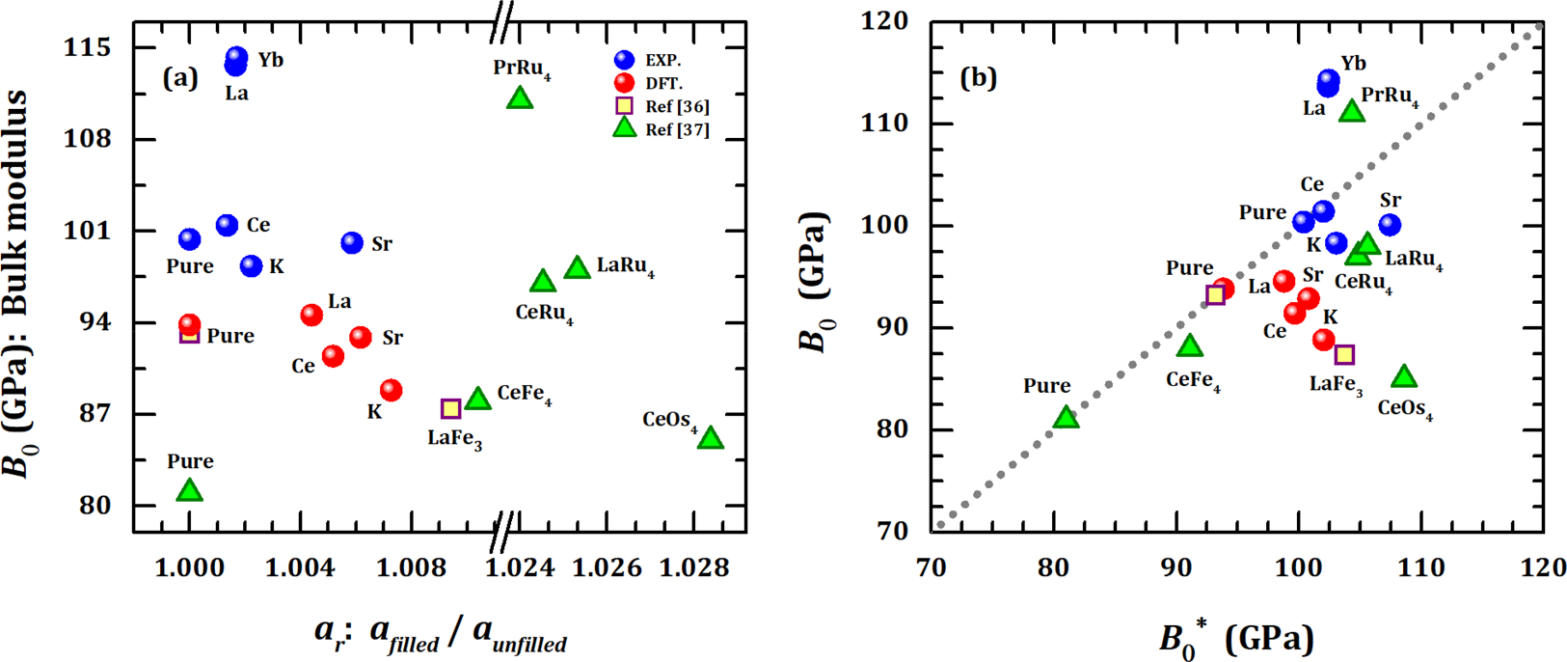
(a) Bulk modulus (*B*_0_) against the relative lattice constant *a_r_* (*a_r_* = *a*_filled_/*a*_unfilled_) for the unfilled Co_4_Sb_12_ (denoted as “pure”) and M_0.5_Co_4_Sb_12_ skutterudites (M = K, Sr, La, Ce, and Yb) investigated. It also includes the *B*_0_ values for filled skutterudites MRu_4_Sb_12_ (M = La and Pr), Ce*T*_4_Sb_12_ (T = Fe, Ru, and Os),^37^ and LaFe_3_CoSb_12_.^36^ (b) Comparison between Bulk modulus calculated from eq 2 (*B*_0_*) and theoretically/experimentally obtained (*B*_0_). The gray dotted line stands for *B*_0_ = *B*_0_*.

A better comparison between experimental and calculated Bulk modulus is shown in Figure 6a, in which this parameter is plotted against the relative lattice constant *a_r_* (*a_r_* = *a*_filled_/*a*_unfilled_), being defined as the ratio between lattice constant of filled skutterudite and that for the unfilled sample. Also plotted in Figure 6a are the Bulk moduli for filled skutterudites MRu_4_Sb_12_ (*M* = La and Pr), CeT_4_Sb_12_ (T = Fe, Ru, and Os),^37^ and LaFe_3_CoSb_12_.^36^ Except for K_0.5_Co_4_Sb_12_ and LaFe_3_CoSb_12_, the lattice expansion, at least, leads to an increase in the Bulk modulus. When K is the filler atom, the general tendency observed for both experimental and theoretical techniques is to reduce *B*_0_ and, therefore, to enhance the compressibility. The Bulk modulus is almost unchanged for the K filling. Significant variations in the *B*_0_ value were observed for La and Yb (*B*_0_ ∼ 114 GPa) as fillers in □Co_4_Sb_12_ (*B*_0_ ∼ 100 GPa). It is also worth mentioning that the La- and Yb-filled samples depict the highest values of *B*_0_ among the antimony-based skutterudites in Figure 6.

**Figure 7:**
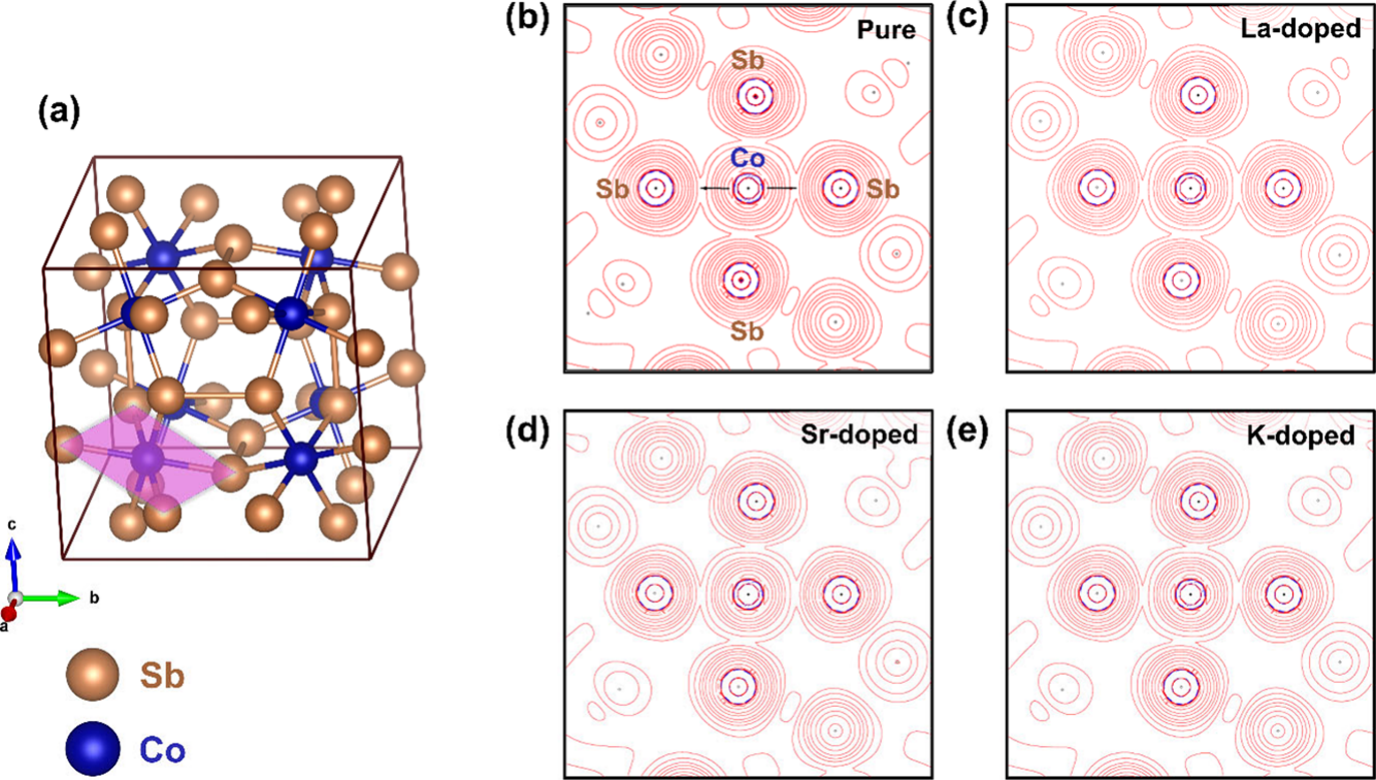
3D structure highlighting the plane of interest for topochemical studies (pink) (a). Laplacian of electronic density isolines of the Co–Sb interaction (b–e).

**Figure 8:**
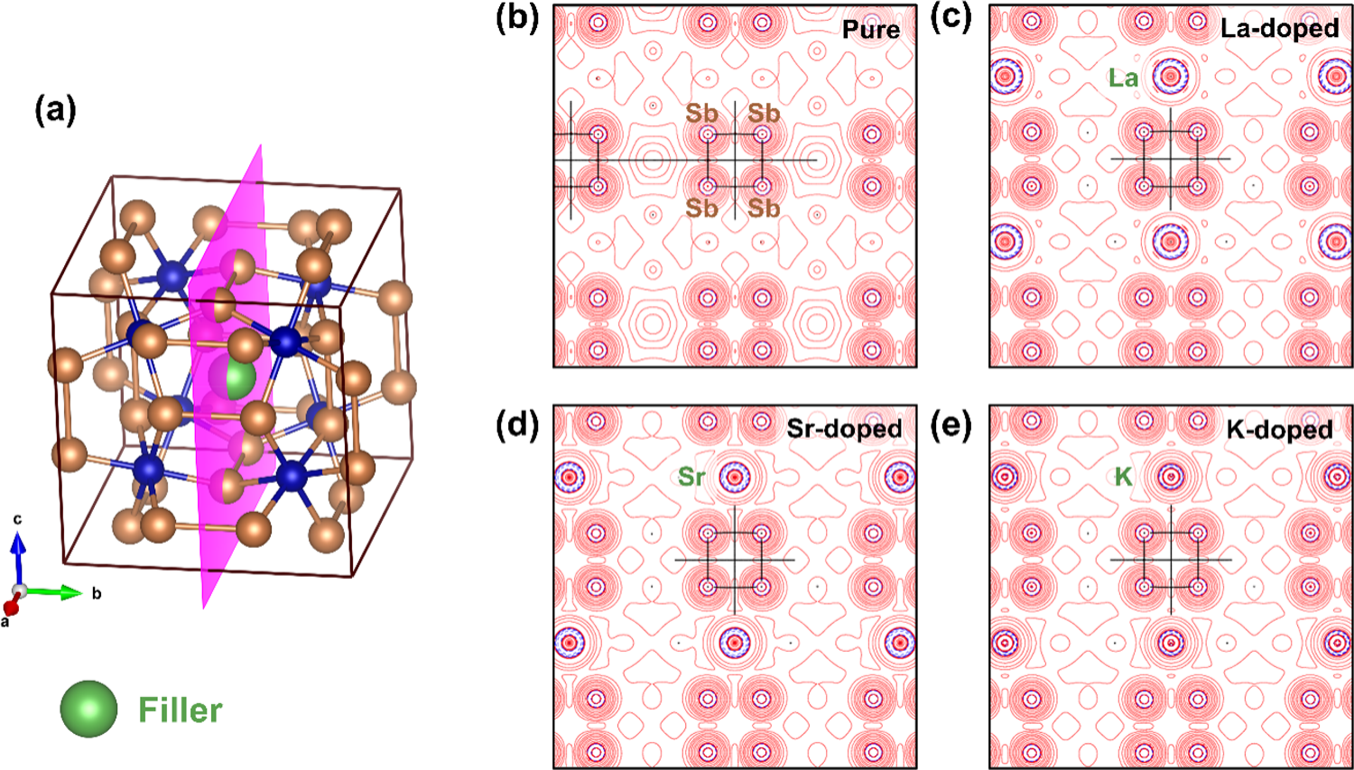
3D structure highlighting the plane of interest for topochemical studies (pink) (a). Laplacian of electronic density isolines of the M–Sb and Sb–Sb interactions (b–e). The [Sb_4_] ring is represented in each plane.

Topochemical analysis of the bond order of the filler site environment was carried out to evaluate the atomic bond variations between the different systems. Figures 7 and 8 exhibit the planes and bonds that have been considered. Table 2 presents the density, Laplacian electron density, bond degree, and |*V*|/*G* ratio.

**Table 2:** Topochemical Parameters of Pure and Doped □Co_4_Sb_12_ Crystals at the Bond Critical Point: Charge Density (ρ(r)), Laplacian of Electron Density (∇^2^ρ(r)), |*V*|/*G* ratio, and Bond Degree (*H*/ρ(r))^a^

composition	bond	ρ	∇^2^ρ	|*V*|/*G*	*H*/ρ
□Co_4_Sb_12_	Co–Sb	0.060	0.066	1.501	–0.275
	Sb–Sb	0.044	0.007	1.833	–0.209
	Sb–Sb	0.052	0.005	1.907	–0.248
K_0.5_Co_4_Sb_12_	Co–Sb	0.058	0.065	1.484	–0.264
	Sb–Sb	0.042	0.010	1.780	–0.199
	Sb–Sb	0.050	0.007	1.864	–0.238
	K–Sb	0.010	0.031	0.832	0.109
Sr_0.5_Co_4_Sb_12_	Co–Sb	0.058	0.064	1.488	–0.264
	Sb–Sb	0.043	0.008	1.809	–0.203
	Sb–Sb	0.050	0.006	1.884	–0.241
	Sr–Sb	0.012	0.037	0.913	0.060
La_0.5_Co_4_Sb_12_	Co–Sb	0.058	0.064	1.485	–0.263
	Sb–Sb	0.043	0.008	1.807	–0.203
	Sb–Sb	0.050	0.007	1.876	–0.238
	La–Sb	0.020	0.035	1.125	–0.061

a *V*: virial potential energy, *G*: kinetic energy density, and *H*: total energy density.

Starting the data evaluation of the pure □Co_4_Sb_12_ system, it was noted that, in a general observation, it presents transient bonds with a tendency leaning slightly toward the ionic character. This can be seen by the low ρ(r) values and positive values of ∇^2^ρ(r). As expected, the Sb–Sb interaction presents ∇^2^ρ(r) smaller and |*V*|/*G* close to 2, which means a less ionic character as compared to Co–Sb bonds.^40^

With the addition of the fillers, in all models, a slight decrease in the values of ρ(r) and an increase in ∇^2^ρ(r) of the Co–Sb and Sb–Sb connections are observed. Our simulation used M = K, Sr, and La as representative situations for the next valence states 1+, 2+, and 3+, respectively. This fact represents a slight disturbance and an increase, even if it is small, in the ionic character of the system as a whole. In addition, another interesting factor is the M**–**Sb interaction around the filler site. For Sr and K fillers, values of 0.012 and 0.010 of ρ(r) and 0.037 and 0.031 of ∇^2^ρ(r) show less localized electrons between the M–Sb nuclei in relation to the Co**–**Sb bonds. In addition, values lower than 1 of |*V*|/*G* and the inversion of the *H*/ρ magnitude (positive) indicate “closed-shell” bonds with ionic/van der Walls interactions. On the other hand, values of |*V*|/*G* greater than 1 and *H*/ρ negative observed in La–Sb indicate an interaction with a covalent incipient character. The *H*/ρ negative also indicates a higher direct bond interaction between the La and the Sb site neighbors. This fact shows that La incorporates different interactions than those incorporated by Sr and K.

The presence of the filler inside the voids generates an internal pressure, which is responsible for the lattice expansion observed under ambient conditions. Then, the Bulk modulus of the expanded structure (*B*_0_*), as compared to the unfilled □Co_4_Sb_12_ (*B*_0|un_), would be approached by *B*_0_* – *B*_0|un_ ≈ *B*_0_′Δ*P*. The variation in pressure Δ*P* can be replaced by −*B*_0|un_Δ*V*/*V*_0|un_, in view of the Bulk modulus definition; Δ*V* represents the volume expansion induced by the filler. Different from the assumption taken by Kraemer *et al.*,^36^ we have considered that Δ*P* has a negative sign since the direction of the pressure field inside the voids is opposite to that in typical experiments of compression:
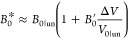
2

In Figure 6b, a comparison between the Bulk modulus as evaluated by eq 2 and those experimentally/theoretically obtained is shown. One may see that a poor agreement is achieved, which means that a simple lattice expansion is not enough to account for the variation of the Bulk modulus in filled skutterudites.

It is clear that a more complex interaction among the fillers and the skutterudite framework takes place in filled skutterudites, which is not accounted by eq 2. An electronic model for this behavior considers that the filler M donates their valence electrons to the conduction band of the skutterudite framework and, therefore, the character of the chemical bonds between the filler and the Sb framework atoms has an ionic character. Such a fact can be observed by the variation in the electronic charge around the [Sb_4_] ring when the fillers enter into the skutterudite structure (see in Table 2). Then, the variation in the Bulk modulus in the presence of the filler elements not only has a component coming from the volume expansion, but the charge localization and ionic character of the filled samples will also contribute to the Bulk modulus changes.

Recently, Hanus *et al*.^33^ studied the local thermal expansion of Co–Sb and Sb–Sb bonds within Yb-filled Co_4_Sb_12_ skutterudite by synchrotron X-ray diffraction. They established, from computational results, the crucial role of the [Sb_4_] ring local structure in predicting the electronic structure changes by means of band convergence. Wang *et al*.^41^ concluded that the filler acts on the A_g_ phonon mode of the [Sb_4_] ring by stretching and compression of the vibration, which affects the Sb–Sb antibonding states. Those states are crucial for the valence band maximum, and therefore, the band gap becomes tunable. In our case, we have observed for K-, Sr-, and La-filled Co_4_Sb_12_ skutterudites that the filler induces a disturbance and an increased ionic character within the lattice. As a result, the high-pressure behavior presented slight changes by means of the Bulk modulus. High-pressure studies are shown to be a promising method to probe the filler effects in skutterudites for thermoelectric applications.

## 4 Conclusions

In this work, we investigated the high-pressure behavior of unfilled □Co_4_Sb_12_ and filled M_0.5_Co_4_Sb_12_ (M = K, Sr, La, Ce, and Yb) skutterudites by synchrotron X-ray diffraction. The skutterudite compounds were also synthesized under high pressure, as we reported previously in refs (11−14). In the pressure range 0–12 GPa, the EOS were obtained and the Bulk moduli (*B*_0_) were estimated for all the filled skutterudites. The Bulk modulus of each composition was estimated and compared to the values calculated by DFT methods. We also estimated the variation of *B*_0_ using a linear approach to predict its values (see in eq 2). We concluded that a simple lattice expansion due to the filler atoms is not enough to describe the experimentally derived *B*_0_ values. Topochemical investigations of the filler site environment were carried out in order to evaluate the bond changes as a function of the filler element. The theoretical results showed a slight disturbance and an increased ionic character when the filler is stabilized inside the skutterudite voids, and therefore, the mechanical properties are indeed affected, as we observed in the Bulk modulus.
